# Saphenous Vein Graft Aneurysm after Drug-Eluting Stent Implantation: Treatment by Covered Stent

**DOI:** 10.1155/2021/2360804

**Published:** 2021-11-05

**Authors:** Kyriacos Papadopoulos, Panayiotis Avraamides

**Affiliations:** Cardiology Department, Nicosia General Hospital, Nicosia, Cyprus

## Abstract

Saphenous vein graft aneurysms (SVGAs) occur as a rare complication of coronary artery bypass graft but increases the risk of morbidity and mortality. Atherosclerosis is considered to be the most common cause of saphenous vein graft aneurysms. Other etiologies include infections, varicosities of vein grafts, and surgical technical issues. These aneurysms usually present as an incidental finding of a mediastinal or cardiac mass on chest radiograph. Symptomatic aneurysms may present with a wide variety of clinical manifestations such as chest pain/angina, shortness of breath, and myocardial infarction. Treatment options of SVG aneurysms include surgery, percutaneous intervention (including vascular plugs, covered stents, and embolization coils), and conservative management. Herein, we describe a case of a saphenous vein graft aneurysm that developed after percutaneous intervention, which has never been described, to our knowledge, in the previous literature. The aneurysm was treated with polytetrafluoroethylene covered stent implantation.

## 1. Introduction

Coronary artery bypass grafting (CABG) has become one of the most common surgical procedures performed across the world since Favaloro first described it in 1967. Aneurysmal dilatation of aorto-coronary SVGs (saphenous vein grafts) was first reported in 1975 by Riahi and associates [1].

Saphenous vein graft aneurysms (SVGAs) occur as a rare complication of coronary artery bypass graft but increases the risk of morbidity and mortality. Atherosclerosis [2] is considered to be the most common cause of saphenous vein graft aneurysms. Other etiologies include infections, varicosities of vein grafts, and surgical technical issues [3–5].

Treatment options include surgery, percutaneous intervention such as such as coil embolization and deployment of a polytetrafluoroethylene- (PTFE-) covered stent and conservative medical management [6, 7]. Herein, we describe a case of a saphenous vein graft aneurysm that developed after percutaneous intervention, which has never been described, to our knowledge, in the previous literature. The aneurysm was treated with polytetrafluoroethylene covered stent implantation.

## 2. Case Report

A 70-year old man with previous history of three vessel coronary artery bypass graft surgery (left anterior descending to left internal mammary artery (LIMA to LAD), SVG to posterior descending artery (PDA), and SVG to obtuse marginal (OM)) was admitted to the Cardiology Department of Nicosia General Hospital with unstable angina. The patient complained of tight precordial pain (lasting for 15-20 minutes), radiating to the left arm, and accompanied by sweating. Other medical problems include type 2 diabetes, dyslipidaemia, and hypertension.

On physical examination, the blood pressure was 115/78 mmHg, the heart rate 87 beats per minute, and the respiratory rate 16 breaths per minute.

His auscultation revealed normal first and second heart sounds with no murmurs. No third or fourth heart sounds were heard.

Electrocardiogram on admission showed normal sinus rhythm with T-wave inversions in leads II, III, aVF.

Chest X-ray was clear, and serial cardiac enzymes were negative.

The transthoracic echocardiogram revealed normal left ventricular size with mildly reduced ejection fraction (LVEF: 45%) and severe hypokinesis of the inferior wall. The aortic valve was trileaflet with mildly thickened leaflets. The mitral valve annulus was calcified with no mitral stenosis or regurgitation.

**Figure 1:**
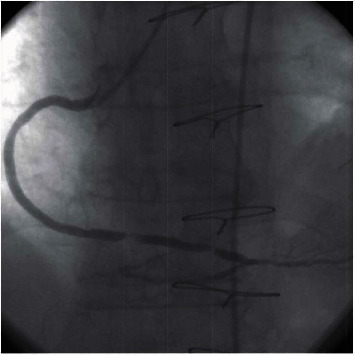
Coronary angiography revealed two consecutive critical stenosis of the SVG to PDA.

The patient underwent coronary angiography which demonstrated total occlusion of mid LAD after giving rise to the first diagonal branch that had a separate severe stenosis at its origin. LCx (left circumflex) was totally occluded from its proximal part after giving off the first obtuse marginal artery that was subtotally occluded at the ostium. RCA (right coronary artery) had a severe stenosis in the proximal segment and was totally occluded at the midportion. The LIMA to LAD graft was patent and flowing into distal native artery. The SVG to OM was occluded ostially, and the SVG to PDA had two consecutive critical stenoses located in the distal segment (Figure 1).

**Figure 2:**
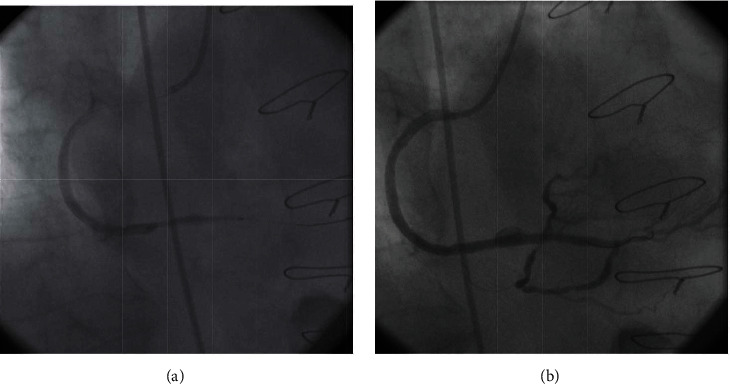
(a) An everolimus eluting-stent was implanted to cover the entire diseased segment of the SVG. (b) Check angiogram showed a focal aneurysm formation at the site of the stent implantation.

**Figure 3:**
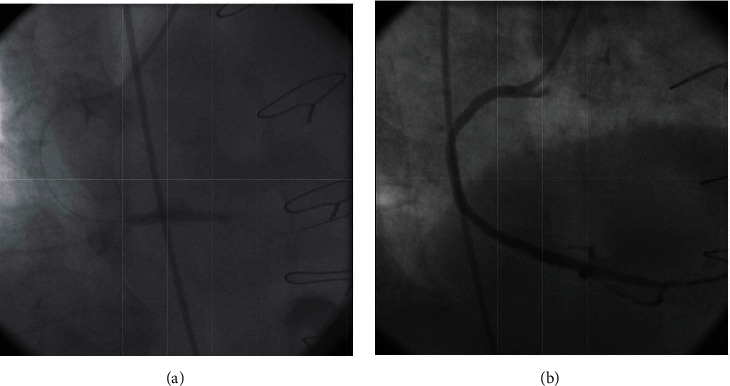
(a) A covered stent was deployed across the aneurysmal segment of the vessel. (b) Completion angiogram showed complete exclusion of the aneurysm.

Ad hoc PCI to SVG to PDA was performed using a Judkin's right 4 (JR4) guiding catheter and a 0.014” balanced middleweight (BMW) guide wire. An everolimus eluting-stent (Xience Pro 4.0 × 33 mm) was directly deployed at the lesion site in the distal segment of the SVG (Figure 2(a)). Subsequent contrast injection revealed a focal aneurysm formation at the site of the stent implantation (Figure 2(b)). The patient maintained a stable hemodynamic condition with a BP = 132/72 and HR = 92. Considering the potential risk of rupture, we decided to seal the aneurysm with a PTFE-covered stent. A Graft Master 4.0 × 26 mm covered stent was deployed within the aneurysmal segment of the stented SVG at an inflation pressure of 10 atmospheres (Figure 3(a)). Postcovered stent angiogram showed total exclusion of the aneurysmal sac with good distal flow (Figure 3(b)). After the procedure, the patient remained hemodynamically stable and a control echocardiogram excluded pericardial effusion. The postoperative period was uneventful, and the patient was discharged after 2 days on 75 mg of aspirin and 75 mg of clopidogrel for at least one year. The patient was planned to have a control coronary angiogram at 6 months.

## 3. Discussion

SVGA is defined as a focal dilation of the vessel to 1.5 times greater than the proximal reference diameter [8].

The etiology of aneurysm formation remains unclear. Late aneurysm formation (i.e., >5 years after CABG) is thought to result from atherosclerotic degeneration leading to structural to weakening of the vessel and subsequent graft dilation [9]. Early aneurysms <12 months after surgery may be related to intrinsic weakness of the vessel wall [10], infections [11], and technical problems.

Although percutaneous coronary intervention-induced aneurysms of the coronary arteries have been increasingly reported over the past few years, formation of SVG aneurysm after stent implantation has never been described. In our case, the development of the aneurysm occurred after everolimus-eluting stent placement. Mechanical factors responsible for aneurysm formation include oversized balloons/stents or high-pressure balloon inflations, all of which can cause dissection and vascular wall injury with weakening and stretching of the vessel [12–14]. After drug-eluting stents implantation, the elution of antiproliferative drugs in combination with the presence of a polymer may result in delayed reendothelialization and incomplete healing, local hypersensitivity reactions, inflammation, and incomplete stent apposition, eventually leading to aneurysm formation [15].

CABG aneurysms can be divided into true aneurysms and pseudoaneurysms. Pseudoaneurysm is a rare postoperative complication, most frequently appearing within the first 6 months of surgery [16]. They usually occur often occur near sites of surgical anastomosis. Potential causes of pseudoaneurysms include surgical technical issues and infections.

True aneurysms commonly occur at the body of the graft [17, 18] and involve the entire vessel wall. They appear as a late complication [15] (>5 years after CABG) and are considered to be atherosclerotic in origin.

These aneurysms usually present as an incidental finding of a mediastinal or cardiac mass on chest radiograph [19–21]. Symptomatic aneurysms may present with a wide variety of clinical manifestations such as chest pain/angina, shortness of breath, and myocardial infarction.

Reported complications of SVG aneurysms include rupture, cardiac tamponade, hemothorax, distal embolization, myocardial infarction, compression of adjacent structures, and fistula formation [8, 22].

The most frequently affected grafts are those directed to the right coronary arteries, followed by the left anterior descending coronary artery.

Most aneurysms can be detected as mediastinal masses on plain chest X-rays. Computed tomography can demonstrate the size of the aneurysm and its relation to surrounding structures. Cardiac MRI is another noninvasive imaging modality with no risk of exposure to ionizing radiation which can define the content and mass effect of SVG aneurysm. Transesophageal echocardiography provides additional information about the size and intraluminal pathology. Two-dimensional echocardiography can provide diagnostic information about the cardiac function [23]. Coronary angiography is definitive in confirming the graft dilatation and can detect other lesions in the native coronary circulation. Intravascular ultrasound (IVUS) has become the “gold standard” for providing critical diagnostic information regarding the morphology, pathogenesis, and management of SVG aneurysms. IVUS is useful to differentiate true aneurysm from pseudoaneurysm which is important in choosing the optimal strategy.

No consensus exists on the optimal treatment of patients SVG aneurysms. Currently, there are no evidence-based guidelines to direct patient management. Treatment options of SVG aneurysms include surgery, percutaneous intervention (including vascular plugs, covered stents, and embolization coils), and conservative management. In asymptomatic patients with small SVGAs (less than 1 cm in diameter), conservative medical management may be reasonable. Operative therapy may include aneurysmal resection or ligation followed by bypass grafting when the native coronary artery requires further revascularization. In patients with other indications for cardiac surgery (multiple territories requiring revascularization or concomitant valve surgery) or mechanical complications, surgical treatment should be pursued. In addition, for patients with graft anatomy not amenable to stent implantation, surgery is the preferred method of treatment. Percutaneous treatment offers an alternative method of management in elderly patients where risk of repeat surgery is very high. Different approaches are used depending upon the anatomic characteristics of the aneurysm and coronary circulation. Interventions utilizing coil embolization [24] or vascular plug insertion [25] are indicated when the affected graft is occluded or the coronary territory is perfused. Covered stent implantation is considered a suitable option when continued antegrade SVG flow is required [6, 26, 27]. In our patient, the SVG was patent, so a covered stent was selected as the preferred treatment.
